# Factors predicting mood changes in oral contraceptive pill users

**DOI:** 10.1186/1742-4755-10-45

**Published:** 2013-09-09

**Authors:** Ghodratollah Shakerinejad, Alireza Hidarnia, Mohammad Esmaeil Motlagh, Khodabakhsh Karami, Shamsoddin Niknami, Ali Montazeri

**Affiliations:** 1 Department of Health Education, Faculty of Medical Sciences, Tarbiat Modares University, Tehran, Iran; 2 Department of Public Health, Faculty of Medicine, Jundi Shapur University of Medical Sciences, Ahwas, Iran; 3 Department of Public Health, Faculty of Health, Jundi Shapur University of Medical Sciences, Ahwas, Iran; 4 Mental Health Research Group, Health Metrics Research Centre, Iranian Institute for Health sciences Research, ACECR, Tehran, Iran

## Abstract

**Background:**

Over 100 million women worldwide are using oral contraceptives pills (OCP) and mood changes were being as the primary reason for OCP discontinuation. The purpose of this study was to determine the prevalence and predicting factors of mood changes in oral contraceptive pills users.

**Methods:**

This was a cross-sectional study of 500 women aged 15–49 years old using low dose (LD) pills attending family planning centers in Ahwaz, Iran. Data were collected via face-to-face interviews using a structured questionnaire including items on demographic, self-efficacy and mood change. Both univarate and multiple logistic regression analyses were performed to assess the relationship between reported mood change and the independent variables.

**Results:**

In all 406 women reported that they did experience OCP side effects. Of these, 37.7% of women (*n* =153) reported mood changes due to OCP use. The results of multiple logistic regression revealed that place of living (*OR =*2.57, 95% *CI =* 1.06-6.20, *p =*0.03), not receiving information on OCP side effects (*OR =*1.80*,* 95% *CI =* 1.15-2.80, *p =*0.009), and lower self-efficacy (*OR =* 0.87, 95% *CI =* 0.80-0.94, *p =*0.001) were significant predictors of mood changes.

**Conclusion:**

The findings from this study indicated that the prevalence of reported mood changes due to OCP use among Iranian women appeared to be consistent with other studies. In addition the findings showed that receiving information on OCP side effects from health care workers and self-efficacy were important predicting factors for mood changes. Indeed implementing educational programs and improving self-efficacy among women are recommended.

## Background

In order to promote women’s health universal access to reproductive health including using the correct methods of contraception is one of the Millennium Development Goals (MDGs) [1]. Oral contraceptive pills are one of the most common methods of contraception and over 100 million women are using oral contraceptives pills (OCP) worldwide [2]. Also there is evidence that more than 84% of women during their life use one of the hormonal methods in order to prevent pregnancy [3]. However, due to side effects almost 50% of new OCP users discontinue using the pills almost six to twelve months after the start. As such ‘mood changes’ and depression considered as being the primary reason for OCP discontinuation [4,5].

Since the introduction of OCP in 1960, many studies have examined the extent to which oral contraceptive pills affected mood and depression. Nevertheless, the results of the research have been inconsistent and there has been little progress in identifying predictors or causes of OCP-related mood changes [6-8]. ‘Mood changes’ refer to one’s general emotional climate, and emotional state at a particular moment or at a particular day [9].

In Iran OCP is the most common method of pregnancy prevention and the prevalence of OCP use is estimated to be 25% [10]. Yet, ministry of health believes this should be increased or at least to be maintained at the current level [11]. Thus, at policy levels to recognize any barrier in using OCP is considered curtail. Ministry of health reports that almost 35% of users usually discontinue the pill after 12 months of start [12]. Perhaps similar to other societies Iranian women also discontinue using OCP due to side effects. The purpose of this study was to determine the prevalence of mood changes in pill users and identify factors that predict mood changes. It was hoped that the study might contribute to the existing literature and provide a framework for the future interventions.

## Methods

### Design and data collection

This was a cross-sectional study. The study was carried out in Ahwaz, Iran during July 2011 to June 2012. A random sample of women aged 15–49 years old attending family planning centers in urban and rural locations were entered into the study. These centers offer free of charge services to women in reproductive ages. Women could register in a nearby center and usually attend these centers monthly. The centers offer OCP for those who wish and at the first inquiry health care workers provide both printed materials and verbal explanations for women. The centers also provide different services including counseling, family planning, and prenatal and postnatal care. The required sample size was calculated on the basis of findings from the Iran Integrate Monitoring Evaluation Survey (IMES) [10]. Women were recruited randomly from the registry list. To be included in the study, respondents had to be 15–49 years old, married and sexually active, using at least one-month LD pills, and being healthily (not having history of mood/anxiety disorders, PMS/PMDD, and use of psychotropic medications). To collect data face-to-face interviews were conducted using a structured questionnaire. A team of trained public health students not connected to the family health services carried out all interviews.

### The questionnaire

A three-part questionnaire especially designed for the study was developed. It contained items on demographic, side effects of pill use, and self-efficacy. These are presented as follows:

1. Demographic information: included recording of age, education, employment, ethnicity, place of living, and duration of OCP use of current formulation.

2. Side effects of oral pill use: First it was asked whether a respondent experienced any side effects due to the current OCP use. Then, if the answer was yes, we asked women to name any side effects they had experienced during OCP use. Respondents were allowed to name as many side effects as they experienced. Also we asked women whether they have received any information on OCP side effects from health care workers in the centers.

3. Self-efficacy: for the assessment of perceived self-efficacy, a six-item condition specific to oral pill self-efficacy scale was used. Items were generated from existing self-efficacy measures [13-15] as follows:

1. If I forget to use my pill, I can follow the instruction.

1. If I receive information on pill use, I can use it correctly

1. If I get sick when using oral pill, I can overcome the problem

1. If I get headache when using oral pill, I can overcome the problem

1. If I experience mood changes when using oral pill, I can overcome the problem

1. If I experience side effects, I can overcome the problem

Response categories ranged from 1 (not at all true) to 4 (exactly true) giving a total score ranging from 6 to 24 where the higher scores indicated higher self-efficacy. Reliability and stability of the item set as assessed by internal consistency and intra-class correlation coefficient (ICC) showed satisfactory results (Cronbach’s alpha coefficient = 0.78 and ICC = 0.84).

### Analysis

Descriptive statistics was used to explore the data. Both univariate and multiple logistic regression analyses were used to assess the association between mood changes and independent variables including age, education, ethnicity, place of living, duration of OCP use, receiving information on OCP side effects, and self-efficacy. Data were analyzed using SPSS, version 16. Statistic significance was set at P < 0.05.

### Ethics

The ethics committee of Tarbiat Modares University approved the study. All participants gave informed oral consent.

## Results

In all 650 women were approached during the study period. Of these 50 women refused and the remaining 600 women agreed to take part in the study. However, due to several reasons including incomplete questionnaires, not using oral contraceptive pills, or not reporting side effects additional 100 women were excluded from the study; giving 500 questionnaires ready for the analysis. The mean age of participants was 27.5 ± 5.6 years, ranging from 15 to 47 years. Most women had primary education (58.2%), and were housewife (85%). The mean self-efficacy score was 12.9 (SD = 2.70) raging from 6 to 24 and overall 94 women (18.8%) indicated that they did not experience any side effects due to OCP use, while the remaining 406 women (81.2%) reported that they were experiencing side-effects. The characteristics of respondents are shown in Table 1.

**Table 1:** The characteristics of the study sample

	**All (*n*** **= 500)**	**Women experiencing OCP side effects (*n*** **= 406)**	**Women not experiencing OCP side effects(*n*** **= 94)**	P-value*
**Age (year)**				
Mean (SD)	27.5 (5.6)	27.81 (5.58)	27.56 (5.42)	0.09
Range	15-47	15-43	16-47	
**Parity (number)**				
Mean (SD)	2.00 (1.18)	2.07 (1.17)	1.84 (0.96)	0.57
Range	0-10	0-7	0-10	
**Duration of OCP use (months**)				
Mean (SD)	25.1 (3.45)	27.75 (6.79)	23.44 (10.03)	0.34
Range	5-55	5-50	5-55	
**Education (number, %)**				0.57
Illiterate/primary	164 (32.8)	129 (31.8)	35 (37.2)	
Secondary	291 (58.2)	240 (59.1)	51 (54.2)	
Higher	45 (9)	37 (9.1)	8 (8.6)	
**Ethnicity (number, %)**				0.84
Arab	334 (66.8)	272 (66.9)	62 (66.0)	
Lor	130 (26)	106 (26.1)	24 (25.5)	
Fars	36 (7.2)	28 (7.0)	8 (8.5)	
**Place of living (number, %)**				0.002
Rural	75 (15)	51 (22.6)	24 (25.6)	
Urban	425 (85)	355 (77.4)	70 (74.4)	
**Self-efficacy score**				
Mean (SD)	12.91 (2.70)	12.74 (2.37)	14.03 (3.02)	0.017
Range	6-24	6-24	6-24	
**Receiving information on OCP side effects (number, %)**				0.001
Yes	293 (58.6)	221 (54.4)	72 (76.5)	
No	207 (41.4)	185 (45.6)	22 (23.5)	

* Comparisons were made between women who did and did not report experiencing OCP use side effects. P-values derived from t-test for continuous data and chi-square for categorical information.

The most important side effects are presented in Table 2. As shown, 37.7% reported mood changes during pill use. In order to assess the relationship between mood changes and independent variables we performed both univariate and multiple logistic regression analyses. For the analysis negative mood changes treated as dependent variable and demographic information, receiving information on OCP side effects from health care workers, and self-efficacy scores were considered as dependent variables. The results obtained from multiple logistic regression analysis indicated that women who reporting living in an urban setting *(OR =* 2.57*, 95% CI =* 1.06-6.20*, p* = 0.03*)*, who did not receive information on OCP side effects *(OR =* 1.80, 95% *CI =* 1.15-2.80*, p* = 0.009*)* and who reported lower self-efficacy scores *(OR =* 0.87*, 95% CI =* 0.80-0.94*, p =* 0.001*)* were more likely to report experience of mood changes. The relationship between mood changes and others variables studied did not show any significant results. The results are shown in Table 3.

**Table 2:** **Side effects of the oral pill use reported by women (*n*** **= 406)**

	**Number**	**%**
**The most important side effects**		
Mood changes	153	37.7
Nausea	65	16.0
Headache	64	15.8
Chloasma (facial pigmentation)	44	10.9
Decrease in sexual desire	17	4.2
Decreased bleeding	14	3.4
Other	49	12.0

**Table 3:** The results obtained from logistic regression for reporting mood changes

	OR* (95% CI)	**P**	OR** (95% CI)	**P**
**Age**	0.99 (0.956-1.028)	0.650	1.00 (0.96-1.04)	0.894
**Self-efficacy**	0.86 (0.804-0.934)	0.001	0.87 (0.80-0.94)	0.001
**Duration of OCP use**	0.99 (0.989-1002)	0.179	0.99 (0.98-1.00)	0.357
**Education**				
Illiterate/primary	1.0 (ref.)		1.0 (ref.)	
Secondary	1.18 (0.754-1.846)	0.469	1.11 (0.681-1.81)	0.673
Higher	1.83 (0.837-3.873)	0.110	2.06 (0.881-4.81)	0.096
**Received information on OCP side-effects**				
Yes	1.0 (ref.)		1.0 (ref.)	
No	2.08 (1.387-3.135)	0.001	1.80(1.15-2.80)	0.009
**Ethnicity**				
Arab	1.0 (ref.)		1.0 (ref.)	
Lor	1.20 (0.758-1.909)	0.433	0.96 (0.59-1.58)	0.896
Fars	2.11 (0.967-4.630)	0.061	1.76 (0.75-4.12)	0.189
**Place of living**				
Rural	1.0 (ref.)		1.0 (ref.)	
Urban	4.98 (1.924-10.021)	0.001	2.57 (1.06-6.20)	0.035

* Derived from univariate multiple logistic regression analysis.

** Derived from multiple logistic regression analysis.

## Discussion

The findings from this study indicated that the prevalence of reported mood changes due to OCP use among Iranian women appeared to be consistent with other studies. In addition the findings showed that receiving information on OCP side effects from health care workers and self-efficacy were important predicting factors for mood changes. The present study confirmed that most women who used oral pill faced several side effects. Women also reported that mood change was the most important side effects they have experienced during pill use. Unfortunately most warning notes on the pill packages do not address or acknowledge about mood changes adequately while this is an important issue for ensuring that women will continue to use pills in order to prevent at least unwanted pregnancies [16,17]. In fact, discontinuity of oral contraceptive pills might jeopardize women’s health in general and reproductive health in particular [18,19]. A study from the USA found that 33% of oral contraceptive pills users discontinued using pills 6 months after the start due its side effects and 33% of these side effects were emotional and behavioral [4]. Similarly a study from UK showed that the incidence of depression and mood changes among the pill users was about 30% [20]. Evidence from Iran suggest that barriers to use of OCP include health concerns, fear of side effects, misinformation, lack of confidence and sexual dissatisfaction [21,22].

At least four explanations for OCP-related mood changes can be identified. A number of studies have explained some biochemical mechanisms for the effects of oral contraceptive pills on women’s mood changes. In fact they believed that the components of pills such as estrogen and progesterone might cause these behavioral changes [23-25]. A number of investigators believed that rumors and speech about side effects of the pills that generally is said by friends, neighbors and the warning notes on the pill packages would lead to this condition [26-28]. However, some researchers demonstrated that although oral contraceptive pills could cause mood changes, they believed that it will stabilize after while [9,29,30]. Finally, a group of investigators argue that oral contraceptive pills have no effects on mood change at all [31,32].

Regardless of whatever the cause may be, the findings from the present study showed that there were significant association between mood changes and education on how to control side effects of pill, self-efficacy and place of living. Women who received education on how to control side effects of pill in family planning centers were more likely to report less mood changes compared to those who did not. In addition, women living in rural areas have reported lower mood changes in comparison with those who lived in urban environments. Perhaps since primary health care is more active in rural areas, women living in rural area receive more advice on oral pill use than women living in cities and metropolitans. Also women who had higher self-efficacy for using pill experienced less mood changes. Indeed women who had higher self-efficacy had more ability for controlling behavioral changes. Studies have suggested that identification of women who have low self-efficacy should be a priority and providers should promote self care ability and self-efficacy related to using OCP [15,33,34]. It is argued that people with low self-efficacy use avoidance coping strategies while people with high self efficacy use problem-focused coping strategies [35,36].

The descriptive nature of this study might be considered as a limitation and the results should be interpreted with caution, although overall the findings from such studies might help to contribute to the topic. In addition, we relied on self-reported information while it seems that measuring mood changes need a valid and reliable instrument. Thus the findings should be interpreted with caution. Finally one should note that due to time constrain we chose a period of one month of OCP use as an inclusion criteria for this study and this may have been a limitation to our study results.

## Competing interests

The authors declare that they have no competing interests.

## Authors’ contributions

GHS was the main investigator, collected the data, carried out the analysis and wrote the article. AH supervised the study and contributed to writing process. MEM and KK were the study advisors. SN contributed to the study design. AM contributed to the analysis, reviewed the first draft and provided the final manuscript. All authors read and approved the manuscript.
